# Autism Diagnostic Assessments With Children, Adolescents, and Adults Prior to and During the COVID-19 Pandemic: A Cross-Sectional Survey of Professionals

**DOI:** 10.3389/fpsyt.2022.789449

**Published:** 2022-04-28

**Authors:** Debbie Spain, Gavin R. Stewart, David Mason, Janine Robinson, Simone J. Capp, Nicola Gillan, Ian Ensum, Francesca Happé

**Affiliations:** ^1^Institute of Psychiatry, Psychology and Neuroscience, King's College London, London, United Kingdom; ^2^Cambridge Lifespan Autism Spectrum Service, Cambridge, United Kingdom; ^3^Bristol Autism Spectrum Service, Bristol, United Kingdom

**Keywords:** autism, autism diagnostic assessment, post-diagnostic support, COVID-19 pandemic, telehealth, innovation

## Abstract

**Background:**

Access to timely high quality autism diagnostic assessments has traditionally been patchy; many individuals wait months, if not years, for an appointment. The onset of the COVID-19 pandemic has likely impacted autism diagnostic services. This study investigated professionals' experiences of, and thoughts about: (1) how autism diagnostic assessments were conducted before the pandemic; (2) adaptations to service provision because of the pandemic; and (3) challenges, risks, advantages and opportunities associated with autism assessments conducted *via* online platforms (telehealth).

**Method:**

Fifty-two professionals, based in different autism diagnostic services and working with children, adolescents and/or adults, completed an online cross-sectional survey in August and September 2020. This comprised demographic questions (about professionals' roles and experiences), and closed and open questions about service provision and telehealth autism assessments.

**Results:**

There was substantial variation in how autism assessments were conducted prior to and during the pandemic; for example, in relation to the number of professionals involved in the assessment and types of structured, semi-structured and unstructured measures used to conduct this. Fifty-two percent of participants (*n* = 27) reported some service disruption (e.g., full closure, substantial reduction in provision, and/or pausing of in person appointments). Waiting times for assessment had become longer for 58% of services (*n* = 30), due to pandemic-related disruption. Six themes emerged from thematic analysis of open responses: (1) the autism diagnostic pathway, pre-pandemic; (2) initial impact of the pandemic on service delivery; (3) conducting autism assessments during the pandemic; (4) working remotely; (5) improving service design and delivery; and (6) post-diagnostic support. Views about the accessibility, validity, and reliability of conducting telehealth autism assessments were polarized. Some participants considered this efficient, flexible, and adequate; others viewed this as unethical and inappropriate. What constitutes good practice in telehealth autism assessments remains unclear, but there is a general openness to using this method (potentially in a hybrid telehealth—in person model), provided rigor and standardization are enhanced.

**Conclusions:**

The pandemic has potentially compounded existing bottlenecks to the autism diagnostic pathway. Future research should seek to improve timeliness, standardization, accessibility and robustness of this pathway, and the validity and reliability of telehealth autism assessments.

## Introduction

Autism spectrum disorder (ASD; hereafter autism) is a neurodevelopmental condition, characterized by social communication differences, repetitive and/or restricted interests, difficulties managing change, and sensory sensitivities (1). Historically, autism was considered rare, with early prevalence rates estimated as 0.04% of the population (2). Yet, increased knowledge about autism, standardization of assessment tools, and broader conceptualisations of core symptoms (i.e., that there is a spectrum of severity, and traits can be viewed categorically or dimensionally) (3) have meant autism prevalence is now estimated as 1–2% (4). While this makes autism one of the most commonly diagnosed neurodevelopmental conditions, there is likely a “missing generation” of adults who did not receive a diagnosis in childhood; indeed, many people are only diagnosed in adulthood (5).

### Autism Assessments—Prior to the COVID-19 Pandemic

There has been an exponential rise in the number of individuals diagnosed with autism (6). Yet, no biological test for autism exists (7), and it is possible that how autism is assessed influences rates of diagnosis (8). In an extensive review of 21 clinical guidelines used to inform the autism diagnostic process for children, adolescents, and adults in the UK, Hayes et al. (7) described substantial variation in suggestions about what an autism assessment should involve (e.g., sources of information, focal topics in a clinical interview), what methods should be used (e.g., inclusion of formal measures), whether multidisciplinary team (MDT) representation was necessary and how professionals should ideally work together using complementary skills, how to deal with diagnostic uncertainty (e.g., for patients scoring around the threshold on a standardized diagnostic measure) and how to resolve differences of opinion about diagnosis (e.g., between MDT professionals). Studies focusing on clinical guidelines for autism assessment in other parts of the world, such as New Zealand (9), the US and Canada (10), have reported similar differences in guidance for professionals. The implication is that there has been variation in autism assessment service provision. Importantly, the implicit assumption underpinning the diagnostic tradition has been that autism assessments take place in person.

How quickly someone is seen for an autism assessment, and diagnosed with this and/or another condition, can also depend on several factors. These include patient-related factors (e.g., age, presence of other neurodevelopmental, or mental health conditions), professional-related factors (e.g., poor general practitioner or teacher knowledge and understanding of autism and related conditions), service-related factors (e.g., whether there is a local diagnostic service, long waiting times), commissioning processes (e.g., funding availability for local or specialist services), and potentially biased or discriminatory practice (e.g., autism being thought of as a condition only affecting boys and men, fewer people from black, Asian and minority ethnic groups being referred for assessment or diagnosed with autism) [for an overview, see systematic reviews by Estrin et al. (11), Tromans et al. (12), and Walsh et al. (13)]. In most instances, the waiting time for an autism assessment is many months, or even years; a situation patients and families/carers find alarming and distressing, and professionals describe as hugely unsatisfactory (14, 15).

### Autism Assessments—The Impact of the COVID-19 Pandemic

The start of the COVID-19 pandemic in early 2020 and resultant stay-at-home mandates, social distancing guidelines and infection control measures, have substantially impacted routine health service provision across clinical specialities (16). Many autism diagnostic services, for example, started to offer assessments *via* telehealth—defined as remote or virtual contact for health-related appointments, such as *via* videoconferencing, telephone or email—, rather than in person, representing a shift from usual practice (17, 18).

Research focusing on telehealth autism assessments, however, is relatively sparse (19). One systematic review by Alfuraydan et al. (17) summarized details of 10 studies (published between 2000 and 2019, i.e., not conducted during a pandemic), in the United States, that evaluated feasibility, acceptability, and/or reliability of telehealth compared to in person autism assessment. The quality of included studies was rated as poor (two studies) or fair (eight studies), with main methodological considerations comprising recruitment of small samples and limited information about sampling frames, inclusion/exclusion criteria and recruitment strategies. Participants were aged between 18 months and 22 years, although two studies did not provide age-related data. The two main telehealth approaches described were: (1) the “real time” method, that involved meeting the patient (and their families/carers) *via* videoconferencing; and (2) the “store and forward” method, that involved families/carers sharing video clips of their child in everyday situations, so as to inform health professionals' diagnostic conclusions. The review findings indicated: (1) most parents and professionals found this easy to use; (2) this could be an efficient use of time, resources, and funding; (3) both telehealth approaches improved access to assessment; (4) service-user satisfaction was relatively high; (5) inter-rater reliability was good when compared with in person assessment; and (6) health professionals liked this way of working, yet many reported a preference for in-person appointments.

### Autism Assessments—The Shift to Telehealth During the COVID-19 Pandemic

The shift to solely using telehealth methods of assessment during the pandemic has potentially raised a range of clinical and practical considerations and complexities. For instance, this necessitates new processes in clinic, and requires professionals to adapt their standard practice, including seeing patients “virtually” from home. This also relies on professionals and patients having access to an internet-enabled device, and adequate space and privacy to meet. Additionally, structured behavioral observation assessments, notably the Autism Diagnostic Observation Schedule−2 [ADOS-2; Lord et al. (20)]—used extensively in diagnostic assessments with individuals across the lifespan—is not validated for use *via* telehealth (21)—meaning that the diagnostic assessment cannot comprise usual components. There is also the question of validity and reliability of a telehealth assessment in a pandemic context (i.e., whether the context substantially alters social communication). These considerations are important, as they can influence the diagnostic outcome, and consequently, options for support for patients and their families/carers.

A very limited number of studies have investigated telehealth autism assessments conducted during the pandemic. One study by Matthews et al. (22) examined the feasibility and acceptability of telehealth autism assessments in 102 people aged between 1.25 and 38 years old seen during Spring and Summer 2020 in the US. The assessment was conducted fully online, and comprised obtaining a developmental history, conducting a behavioral observation assessment, assessing adaptive functioning and intellectual ability, and viewing home videos. Diagnosis was determined in 91% of patients seen, with only nine (seven males, two females, between ages 3–11 years old) asked to attend for further in-person assessment. Findings indicated that patients, families, and health professionals endorsed high levels of satisfaction and acceptability. A second study conducted in Australia (23) used mixed-methods (an online survey with 72 participants and follow up interviews with 25 participants) to investigate autistic adults' (aged 21–76 years old), parents'/carers' and health professionals' perspectives about telehealth assessments conducted between March and September 2020. Overall, findings suggested that telehealth can be convenient, straightforward and satisfactory for some. However, this could also incur complexities, including IT-related practical problems (e.g., poor internet connection, difficulty seeing patients if logging on from a smartphone rather than a computer), and issues with engagement. Some health professional participants also highlighted concerns about assessing subtle autism behaviors, needing to adapt standard practice, and feeling less confident about reaching a diagnostic conclusion. A third study by Wagner et al. (24), conducted in the US between June and November 2020, investigated 202 MDT professionals' experiences of conducting autism assessments with toddlers, *via* telehealth, prior to and during the pandemic. Overall, they found that substantially more professionals had started doing telehealth autism assessments (78% of the sample had used this during the pandemic vs. 6% prior to the pandemic), resulting in changes to the core battery of measures used to inform the assessment (e.g., less use of standardized observational methods). Overall, ~60% of participants reported feeling confident to assess and diagnose autism remotely. However, as in the study by Gibbs et al. (23), and Matthews et al. (22), there were some consistent problems identified that could impact on the diagnostic process, including technological challenges, poor internet access, environmental challenges (e.g., that the home environment did not seem a suitable place to assess patients) and difficulties with engaging families/carers and/or patients.

#### Study Rationale and Aims

The suddenness of the pandemic means autism diagnostic services have been forced to rapidly change traditional ways of working (19, 25). To keep services going, professionals have had to do what seemed best or pragmatic in the circumstances (26). There are, however, no evidence-based guidelines outlining what a good quality telehealth diagnostic assessment *could* and *should* comprise. Moreover, returning to work with personal protective equipment (PPE) and altered working conditions also likely impacted the diagnostic process for many professionals. Taken together, this is potentially concerning, as it could result in even less standardization in how autism assessments are conducted [highlighted in the review by Hayes et al. (7)], and thereby, affect diagnostic conclusions and conversion rates (the number of patients diagnosed with autism vs. the number of patients seen for assessment), and options for support and intervention. While some prior research has examined use of telehealth diagnostic methods, studies have predominantly taken place before the pandemic; a somewhat different context to the present day.

The aim of this study was to better understand how autism diagnostic services have adapted usual practice since March 2020 (i.e., the onset of the pandemic disrupting health services), to examine what professionals think about these adaptations and how confident they feel about assessing patients in this way, and to explore how service provision may be further improved, so that patients and their families/carers can access timely, good quality, reliable and evidence-based assessment. Building on the extant literature, this study explored professionals' experiences of, and perspectives about: (1) how autism diagnostic assessments were conducted prior to the pandemic across settings and services; (2) adaptations to service provision because of the pandemic; and (3) challenges, risks, advantages, and opportunities associated with using telehealth for these assessments.

## Methods and Materials

The research team included autistic and non-autistic researchers, working in clinical practice (several autism services) and/or within a university research department.

### Study Design

The present study reports cross-sectional data from an online survey conducted *via* Qualtrics (www.qualtrics.com) in August and September 2020, as pandemic/lockdown restrictions were somewhat eased in many countries (e.g., social distancing was in place, but non-essential shops were open). Ethical approvals for this study were obtained *via* [King's College London (PNM REC REF HR-19/20-17744). All participants gave informed consent and were asked for permission to use anonymised quotes for dissemination.

### Participants

Inclusion criteria for the study were: (1) MDT professionals (e.g., representing psychiatry, psychology); (2) using English sufficiently proficiently to complete an online survey; (3) involved in conducting autism assessments, with children, adolescents, or adults; and (4) in any country or setting. Potential participants were recruited *via* gatekeepers at health organizations and universities, word of mouth, social media, and the Autistica Network (a UK network of autistic people, family members and professionals, who have consented to contact about research with no obligation to participate).

Fifty-two MDT professionals, based in the UK (*N* = 40, 77%), Europe (*N* = 4, 8%), US (*N* = 5, 10%), Argentina (*N* = 2, 4%), and Australia (*N* = 1, 2%) participated (see Table 1). Six professional disciplines were represented, including clinical and educational psychology, occupational therapy, psychiatry, nursing, and social work. Participants worked across inpatient, outpatient and community health services, the criminal justice system (CJS) and in education. Thirty-eight (73%) of participants worked in public services, three (6%) worked in independent practice and 11 (21%) worked in both sectors. Most worked with adults (*N* = 31, 60%), 16 (31%) with children and adolescents, and five (10%) with people across the lifespan.

**Table 1:** Participant professional demographic characteristics.

		**Whole sample (*****N*** **=** **52)**	
Profession*	Clinical psychologist	31	59.6%
	Occupational therapist	6	11.5%
	Academic researcher	5	9.6%
	Assistant psychologist	4	7.7%
	Psychiatrist	3	5.8%
	Nurse	3	5.8%
	Educational psychologist	1	1.9%
	Social worker	1	1.9%
	Operational service manager	1	1.9%
Years of experience	Up to 4 years	11	21.2%
conducting diagnostic	5–9 years	13	25.0%
assessments	10–14 years	11	21.2%
	More than 15 years	17	32.7%
Work setting	Independent practice	10	19.2%
	Community mental health team	7	13.5%
	autism team	6	11.5%
	Children's health center	6	11.5%
	University health center	3	5.8%
	Private hospital	2	3.8%
	Tertiary service	2	3.8%
	School	1	1.9%
	Criminal justice system	1	1.9%
	Early intervention team	1	1.9%
	Inpatient setting	1	1.9%
	University	1	1.9%
Age of patient group	Children and adolescents (<18)	16	30.8%
	Adult (18+)	31	59.6%
	Lifespan	5	9.6%

* *Participants could endorse more than one professional discipline*.

### Materials

The authors developed the online survey used in this study through conversations with autistic people who had received a diagnosis before or during the pandemic, their parents, health professionals and researchers. Two autistic adults provided feedback on the content and useability of the final survey used.

The survey comprised three sections. The first section pertained to participants' professional demographic characteristics (e.g., their clinical discipline, years of experience with autistic populations, the type of clinical service they work in). The second section pertained to clinical service norms prior to the pandemic (e.g., monthly autism referrals to their service, nature of a diagnostic assessment). The third section pertained to the impact of the pandemic on clinical practice (e.g., service and waitlist closures, adaptations to assessments so that these are COVID-19 safe, clinical judgement concerns) and professionals' thoughts on how autism diagnostic service provision can be enhanced and innovated moving forward. A combination of multiple-choice and open-text responses were used throughout the survey. See Supplementary Materials 1 for full survey questions.

### Procedure

The online survey was accessible for participants to complete on any internet-enabled device for up to 7 days after first logging on. Informed consent was obtained at the first stage of the survey. Survey submission was anonymous, but participants could opt in to a prize draw. The survey took ~15–20 min to complete.

### Analysis

Data pertaining to professional demographic characteristics, and descriptions of service-related factors were summarized descriptively. While professional demographic characteristics were collected to contextualize the sample, responses from participants were analyzed as one group. Qualitative data were analyzed thematically, and involved: (1) becoming familiar with the data; (2) generating initial codes; (3) searching for themes; (4) reviewing tentative themes; (5) labeling themes; and (6) summarizing the data [Braun and Clarke (27), p. 87]. Open ended (free text) responses were reviewed by DS Initial codes were highlighted and categorized consecutively, with labels assigned to themes and subthemes. To enhance rigor, fifty percent of qualitative data were reviewed by DM and codes and themes were compared. The research team sought to be reflexive during survey development and data analysis (28). This included discussing topics such as personal perspectives about the autism diagnostic process, experiences of working in different clinical and academic settings (and meeting people with a range of neurodevelopmental conditions across the lifespan), measures perceived to enhance the robustness of a diagnostic conclusion, ways in which clinical complexity was conceptualized and ideas about the utility and appropriateness of telehealth.

## Results

**Figure 1:**
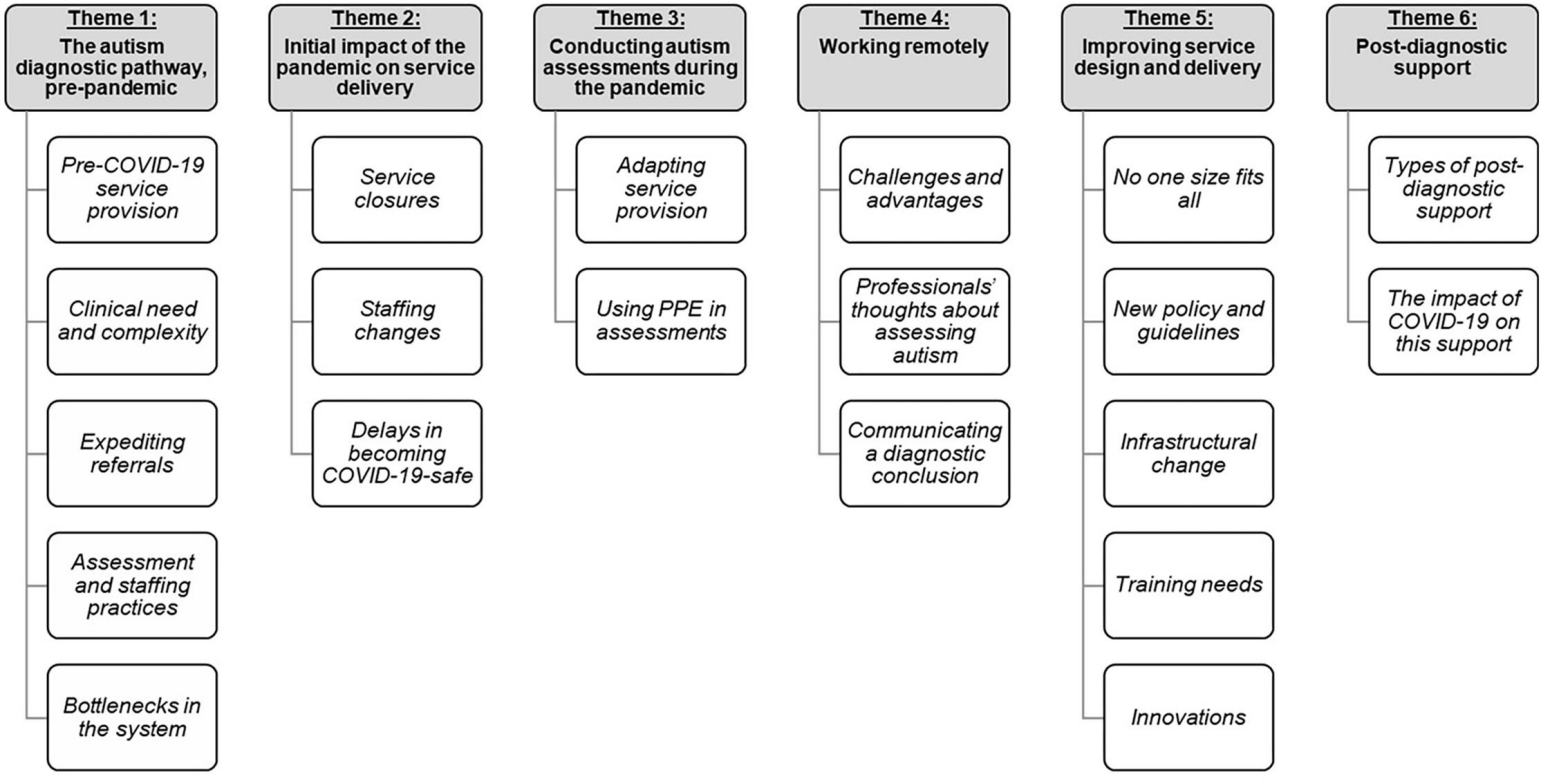
Overview of themes and subthemes.

Thematic analysis of qualitative data suggested there were six themes: (1) the autism diagnostic pathway, pre-pandemic, (2) initial responses to the pandemic, (3) conducting autism assessments during the pandemic, (4) working remotely, (5) improving service design and delivery, and (6) post-diagnostic support (see Figure 1). Additional quantitative data collected from the survey are provided to further contextualize these themes.

### Theme 1: The Autism Diagnostic Pathway, Pre-pandemic

The first theme pertained to participants' clinical experiences prior to the pandemic, including how they assessed autism. This theme includes five subthemes: (1) context of their service, (2) increasing levels of patient clinical need and complexity, (3) whether they expedited referrals and the reasons why, (4) the methods used to assess autism; and (5) whether there were any bottlenecks in the system.

#### Theme 1, Subtheme 1: Pre-COVID-19 Service Provision

Before the pandemic, the number of monthly referrals received by services varied; 42% of services received fewer than 25 referrals, whereas 4% had more than 100. Fifty participants (96%) received referrals from health professionals, 25 participants (48%) also accepted self-referrals and referrals from education and one participant (2%) received criminal justice system (CJS) referrals. Waiting times for assessment ranged from <1 month (4% of services), through to at least 1 year (40% of services).

Professional disciplines represented at services included Psychology, Medicine, Psychiatry, Nursing, Occupational Therapy, Speech and Language Therapy, Physiotherapy, and Art Therapy. Some also employed unqualified staff, including Family Navigators, Peer Support Workers, and Neurodevelopmental Workers.

#### Theme 1, Subtheme 2: Clinical Need and Complexity

Numerous participants reported that patients presented with increasingly high levels of clinical need and complexity; “*referrals are [becoming] more complex, with many people with mental health issues being referred now as well.”* Referrals had become more common for patients with complex backgrounds (e.g., trauma, attachment issues, multimorbidity), a history of inpatient admissions, and/or who had failed to respond to standard pharmacological and psychological intervention approaches.

#### Theme 1, Subtheme 3: Expediting Referrals

Participants were asked about reasons for expediting autism diagnostic assessments. These responses were categorized in terms of: (1) clinical factors; (2) involvement with other services; (3) educational or occupational circumstances; and (4) systemic factors (see Table 2).

**Table 2:** Reasons for expediting referrals.

	**Example of reason in practice**
Clinical factors	Symptoms and presentation (e.g., complexity, multimorbidity)
	Risk (e.g., high risk to self, others, safeguarding concerns)
	Specific clinical population (e.g., Looked After Children, gender dysphoria)
Involvement with other services	Mental health service involvement (e.g., an inpatient on an acute psychiatric ward, at risk of an inpatient admission, diagnostic clarity required to formulate and plan treatment for someone known to mental health services)
	Already seen by partner ADHD service
	Contact with CJS (e.g., current court case, involvement with counter-terrorism organizations)
Educational or occupational circumstances	Education-related concerns (e.g., school placement breaking down, excluded or high risk of exclusion from school, planning to leave university prematurely as unable to cope)
	Pressing issues at work [e.g., involved in a work tribunal, (risk of) job loss]
	Military background (e.g., a veteran, parents in the forces)
Systemic factors	Unsettled social circumstances (e.g., homelessness, imminent loss of accommodation)
	Transition points (e.g., house move, step up to adult services)
	As a result of existing relationships with professionals at the organization (e.g., member of staff, personal relationship with health professionals)

#### Theme 1, Subtheme 4: Assessment and Staffing Practices

How assessments were conducted before the pandemic varied substantially. Eleven participants (14%) conducted these alone, two (40%) worked jointly with one colleague and 12 (23%) collaborated with two plus colleagues. Assessment duration varied from <4 h (*N* = 8, 15%), 4–7 h (*N* = 16, 31%) and 7 h plus (*N* = 24, 46%). Table 3 outlines the frequency of use of different methods of assessment before March 2020.

**Table 3:** Methods of autism assessment used pre-pandemic.

		**Whole sample (*****N*** **=** **52)**	
Structured	ADOS-2	30	57.7%
autism	ADI-R	30	57.7%
assessment	AAA assessment	9	17.3%
	3Di	7	13.5%
Clinical	Family interview	44	84.6%
interview	Clinical interview	43	82.7%
Cognitive	Intelligence (IQ) test	17	32.7%
assessment	Neuropsychological assessment	14	26.9%
	Functional assessment	12	23.1%
Questionnaires	Autism self-report questionnaires	30	57.7%
	Autism informant-rated questionnaires	24	46.2%
	Mental health questionnaires	10	19.2%
Other	Sensory assessment	11	21.2%
assessments	Physical health assessment	7	13.5%

*Participants could select more than one option. ADOS-2, Autism Diagnostic Observation Schedule-2; ADI-R, Autism Diagnostic Interview-Revised; AAA, Adult Asperger Assessment; 3Di, Developmental, Dimensional and Diagnostic Interview*.

#### Theme 1, Subtheme 5: Bottlenecks in the System

Participants described bottlenecks to the smooth running of the autism diagnostic pathway. Summing up a commonly reported view, one participant remarked their service was “*not able to meet the demand of [their] community in a timely way,”* due to “*long waiting times”* and “*demand outstripping capacity.”*

Poor knowledge and understanding of autism from health professionals in primary and secondary care resulted in “*inappropriate referrals or poor-quality referral information.”* This potentially contributed to avoidance of “*responsibility for providing [commissioned] support”* by mainstream services. Lengthy lags between referral and assessment could mean “*information collected at screening [was] no longer up to date.”*

Administrative problems, including “*lack of admin support,” “scheduling challenges,” “high DNA rates,”* and “*[limited] access to suitable rooms,”* were common. These were compounded by logistical considerations, such as “*working in multiple sites,” “significant traveling times,” “lengthy assessments”* and inflexible timetables.

Clinically, the lack of “*systematic triage”* and “*specific protocols for adults”* could affect the assessment. Participants said it was difficult to get the “*balance right between [obtaining] information needed to make a meaningful and robust diagnostic decision and completing as many assessments as we can.”*

Several participants described workforce-related issues, including a lack of both junior and senior staff, poor professional relationships, “*staff changes and sickness”* and “*difficulties trying to keep all needed [clinical] disciplines involved.”*

The lack of commissioned post-diagnostic support was also mentioned by most participants (see Theme 6).

### Theme 2: Initial Impact of the Pandemic on Service Delivery

The second theme pertained to the initial impact of the pandemic on autism services, and how this impacted service delivery. This theme includes three subthemes: (1) how many services experienced a closure, (2) staffing changes due to re-deployment and COVID-19 sickness, and (3) delays in becoming COVID-19-safe.

#### Theme 2, Subtheme 1: Service Closures

The start of the pandemic (defined in the study as March 2020), and introduction of stay-at-home mandates and social distancing measures, resulted in 27 services (52%) closing temporarily. Of these, six (12%) had closed (i.e., were not seeing patients) for up to 4 weeks, three (6%) had closed for between 4 and 8 weeks, four (14%) for between 8 and 12 weeks, five (10%) for between 12 and 16 weeks, and two (4%) for 16 weeks or longer. Six services (12%) remained closed at the time of study participation (August and September 2020). Waiting lists had also closed, with two services (4%) ceasing to accept referrals for 4 weeks, three (6%) for 4–8 weeks, one (2%) for 8–12 weeks, two (4%) for 12–16 weeks, and three (6%) for at least 16 weeks. Four participants (8%) worked in services that had not conducted assessments since the pandemic onset, and one (2%) was only just preparing to restart. Altogether, 30 services (58%) now had a longer waiting time for assessment.

#### Theme 2, Subtheme 2: Staffing Changes

One participant said there had been a “*threat of redeployment to cover essential Trust [health organisation] services,”* resulting in a period of limited activity. Additionally, some professionals had tested positive for COVID-19 and so were off sick, potentially long-term. Others could only work from home (e.g., due to shielding), “*reducing [service] response capabilities.”*

#### Theme 2, Subtheme 3: Delays in Becoming COVID-Safe

Participants described a lack of clarity about governmental guidance, as well as “*continuing changing protocols”* about how best to deliver services. Alongside this, some organizations had swiftly agreed the use of videoconferencing platforms, whereas others had taken substantial time to agree policies for this. One participant highlighted the organization they worked for did not understand that “*doing a diagnostic assessment for a communication disorder via phone and video call is mostly inadequate, leading to issues with accuracy, drop out, stress for staff and patients, and increasing waiting times.”*

### Theme 3: Conducting Autism Assessments During the Pandemic

The third theme pertained to the steps taken to adapt in-person assessments to be more COVID-safe. This theme includes two subthemes: (1) adapting service provision by implementing telehealth assessments, and (2) issues around the use of PPE.

#### Theme 3, Subtheme 1: Adapting Service Provision

Forty-four services (85%) had adapted standard diagnostic processes due to the pandemic. Eighteen services (35%) had offered some in-person appointments, and a further eight (15%) were preparing to at the time of the study (prior to the second wave, in England).

Most services had started collecting background information “*via telephone and video apps.”* Clinical interviews with patients and behavioral observation assessments (e.g., the ADOS-2) had initially been postponed, with some services then opting to do these online and others requiring patients to attend in person when feasible. Some participants reported they needed “*extra information”* beyond what was usual.

By the time of the study, most services were offering assessments fully *via* telehealth, or *via* a blended approach incorporating telehealth and in-person meetings. Adaptations to in-person appointments included: (1) shortening these; (2) limiting how many people could attend; (3) asking patients and families/carers to complete health screens; (4) wearing PPE (see subtheme 3.2); (5) employing social distancing measures; (6) meeting outside; and (7) using materials that could be sanitized or discarded.

Numerous participants talked about the ADOS-2—a mainstay component of diagnostic assessments—not being validated for use remotely, or with PPE. Services had adopted different solutions to this. Some carried out an ADOS-2 with PPE, nevertheless. Others were using “*amended [unvalidated] versions of the ADOS online”* to obtain (unscored) “*qualitative information,”* or they had developed “*a new tool to use remotely”* comprising a “*battery of observational tasks.”* Few services had arranged for professionals to be trained up in the BOSA [the Brief Observation of Symptoms of Autism assessment (29); a proxy to the ADOS-2, requiring facilitation by an adult].

Concerns about the lack of validated instruments and consensus about best practice for conducting assessments during pandemic conditions were frequently cited. One participant remarked; “*my impression is that everyone is managing this differently and there is little evidence to suggest which assessment method, via video link, is any more valid or efficacious than any other.”*

Several participants reported a change in staff allocation, either buddying up with a colleague to see patients when assessments had previously been conducted by sole practitioners, or conversely, having one professional doing the assessment alone. A handful of services reported some tasks—in particular, obtaining the developmental history—were now being conducted by less experienced staff (e.g., assistant psychologists and clinical trainees).

Adaptations to service models appeared to contribute to a lack of parity in service provision, with a knock-on effect on waiting times. One participant reported difficulty “*getting interpreters,”* meaning people who use English as a second language had to wait longer. Age also seemed a relevant factor, as highlighted by one participant, “*our service has shifted to providing telemedicine-based evaluation services, with a focus on young children. As a result, the wait list is shorter for young children to be seen via telemedicine. The wait list is longer for older children who are more likely to require more in-depth, in-person services.”* Conversely, another participant said their service had “*mostly kept going with older children and adults.”* Different child and adolescent services seemingly followed different guidelines. Patients presenting with increased risk to self or others, and “*complex diagnostic cases”* also waited longer.

#### Theme 3, Subtheme 2: Using PPE in Assessments

Some participants had regularly worn face masks, visors, and/or safety glasses. This was considered imperative if meeting patients in person; “*there is no way to get around this—physical safety is more important than the assessment at this time.”*

On the other hand, PPE could be “*off-putting,” “intimidating,”* and “*anxiety-provoking”* for patients, and sensorily uncomfortable. Remaining masked for extended periods could be challenging, resulting in appointments needing to be shorter, and thereby, becoming more resource intensive. One participant said, “*many of our young people cannot tolerate wearing it or lack the level of functioning to understand why they have to wear it... [they] dislike others wearing it and have been ripping aprons and masks off staff ….”*

PPE potentially impacted validity and reliability of autism assessments, as this could “*alter the nature of social interaction,” “affect rapport,” “make it more difficult to evaluate non-verbal communication”* and “*observe social communication differences.”* PPE might result in people behaving “*differently;”* this was crucial, as “*what is normal [social interaction] through PPE?”* is not established. PPE could cause bi-directional difficulties between patients' and professionals' understanding and interpretation of the others' social behavior, “*throwing up questions about whether it is a skill deficit or worsened because of the equipment.”* Echoing several participants' comments, one said they “*[did] not feel comfortable that I could reach a safe diagnostic decision in PPE where so much non-verbal communication is lost.”*

Several participants, including professionals who reported being autistic, identified practical difficulties they had encountered personally, including describing that “*PPE causes me a lot of sensory issues as an autistic person,” “it's hot and uncomfortable,” “restrictive,” “tiring,”* and “*physically uncomfortable for me as a provider. When I leave the hospital, my jaws and ears hurt … my skin is irritated.”*

### Theme 4: Working Remotely

The fourth theme pertained to adapting to a remote work environment. This theme includes three subthemes: (1) the challenge of adapting to and conducting telehealth assessments from a home environment, (2) professionals' thoughts about conducting assessments remotely, and (3) their confidence in reaching and communicating diagnostic conclusions to patients without meeting them in person.

#### Theme 4, Subtheme 1: Challenges and Advantages

**Figure 2:**
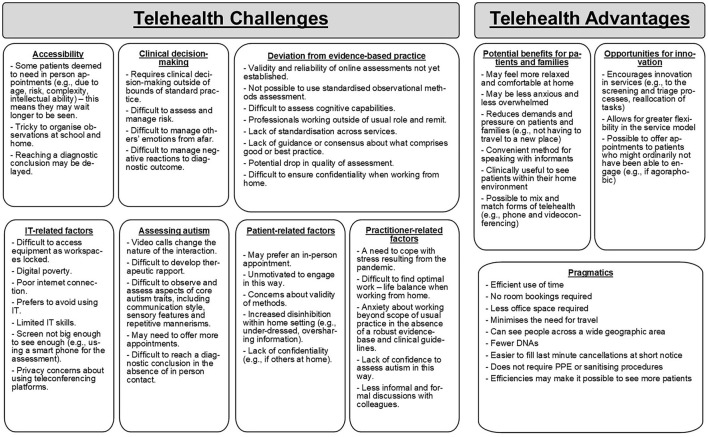
Challenges and advantages to using telehealth for autism assessments.

Participants identified challenges to, and advantages of, conducting telehealth assessments. More challenging aspects of telehealth assessments were categorized into: (1) accessibility; (2) IT-related factors; (3) deviation from evidence-based practice; (4) clinical decision-making; (5) assessing autism; (6) patient-related factors; and (7) professional-related factors (see Figure 2). Conversely, advantages were categorized into: (1) pragmatics; (2) potential benefits for patients and their families; and (3) opportunities for innovation (see Figure 2). There was a lack of consensus about whether advantages outweigh the challenges of this approach, with many participants reporting a need to consider these per patient.

#### Theme 4, Subtheme 2: Professionals' Thoughts About Assessing Autism

Participants rated their degree of confidence in conducting telehealth assessments on a five-point Likert scale. Confidence ranged from “not at all” (*N* = 3, 6%), through to “a lot,” with 33 (64%) participants feeling at least “quite a bit” confident. Within the qualitative data, opinions about telehealth assessments were polarized.

Many participants said telehealth had “*worked really well, and for more ‘straightforward' diagnostic assessments [has] been very positive.”* Others said this seemed appropriate with “*older children/young people where the evidence from parents and school has been strong,”* and that videoconferencing is “*good enough where autism is unlikely.”* Telehealth had also allowed patients to “*show us things in their homes (collections / interests etc.) that they would otherwise not have been able to”* as well as “*the interaction between the parent and child in the comfort of their home.”*

**Figure 3:**
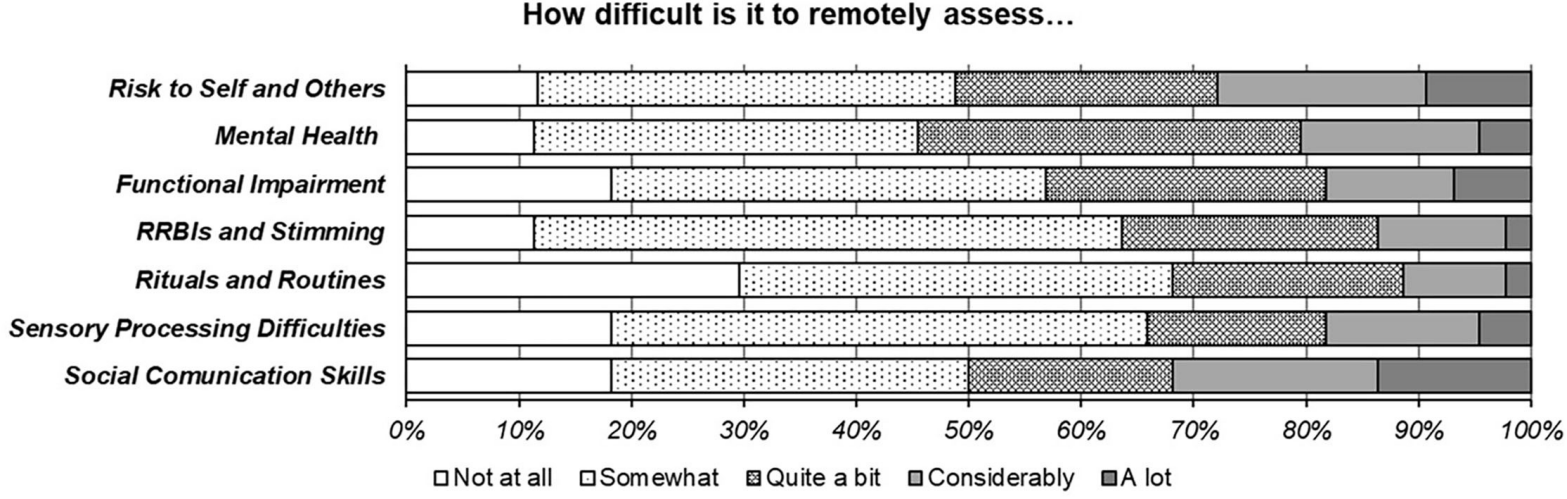
Assessment of symptoms remotely.

Difficulties with assessing some symptoms were also reported by participants (see Figure 3). Taken together, participants felt remote assessment could render it more challenging to “*see the impact of sensory / communication difficulties,”* gain “*a sense of their [patients'] interaction outside of their home,”* observe “*every day interaction such as negotiating toilet breaks or whether they want another cup of tea and can explain how they like it,”* and assess “*eye contact,” “gestures,”* and “*what they are doing with their lower body/hands at times.”* Others mentioned it is “*challenging to evaluate children who are unfamiliar with interacting over a screen … difficult to build rapport”* and “*get a sense of reciprocity.”* Several participants felt “masking” (of autistic traits) may obscure assessment. “*Nuances of social interaction”* could be less clear, and videoconferencing interaction could cause “*conversational asynchrony in the absence of social skills deficits.”* One participant highlighted that this way of working affects “*understanding [of] what the child is responding to in their environment and what you cannot see.”*

Remote assessment of some sub-groups was, on average, deemed more complex (e.g., young and/or overactive children, adults without informants, people with a learning disability or with complex presentations, or clinical symptoms of paranoia, social anxiety, personality disorder, and/or with limited verbal language). Participants mentioned that non-autism features could be challenging to assess remotely, such as “*differential diagnosis”* and “*risk.”*

Concerns were raised about whether it is possible to see “*all the issues” via* telehealth, in particular domestic abuse, if someone is substantially underweight, poor hygiene, or self-neglect. Alongside this, it could be difficult to adequately support patients if they seemed disinhibited, distressed or had started to dissociate.

Some participants reported staunchly negative views about telehealth, describing this as “*inappropriate,” “non-sensical … negligent,”* and “*unethical.”* One participant said “*remote ASD [autism spectrum disorder] assessments have no validity. They do not meet recognized practice standards for diagnostic assessment … Diagnostic assessment is an assessment of social interaction and communication.”* Reflecting several views, another participant noted “*the assessment is much more heavily reliant on the client's report of their difficulties, [rather] than actual observation of difficulties when in the room with them. This makes it more difficult if the client has limited insight into their difficulties, is poor at understanding what you say, or poor at reporting. Alternatively, a bright client, who is heavily invested in the diagnosis, has researched the diagnosis at length and knows ‘how to answer the questions' is likely to give a better presentation—but may be more easily able to influence the outcome—whereas seeing them in the room, gut feeling, interaction, their response to interaction gives clues about the likelihood of them having ASD rather than social issues stemming from trauma or attachment.”*

Summarizing the juxtaposition participants found themselves in, one said “*some clients who present strongly with ASC [autism spectrum condition], for whom a comprehensive background history is available (e.g., ADI-R), a remote assessment via these platforms is very possible. For others, and perhaps the majority, this is not possible. This is especially the case when [the] presentation is complex, and there are other hypotheses about the root of the clients' areas of difference (e.g., developmental trauma, acquired brain injury).”*

**Figure 4:**
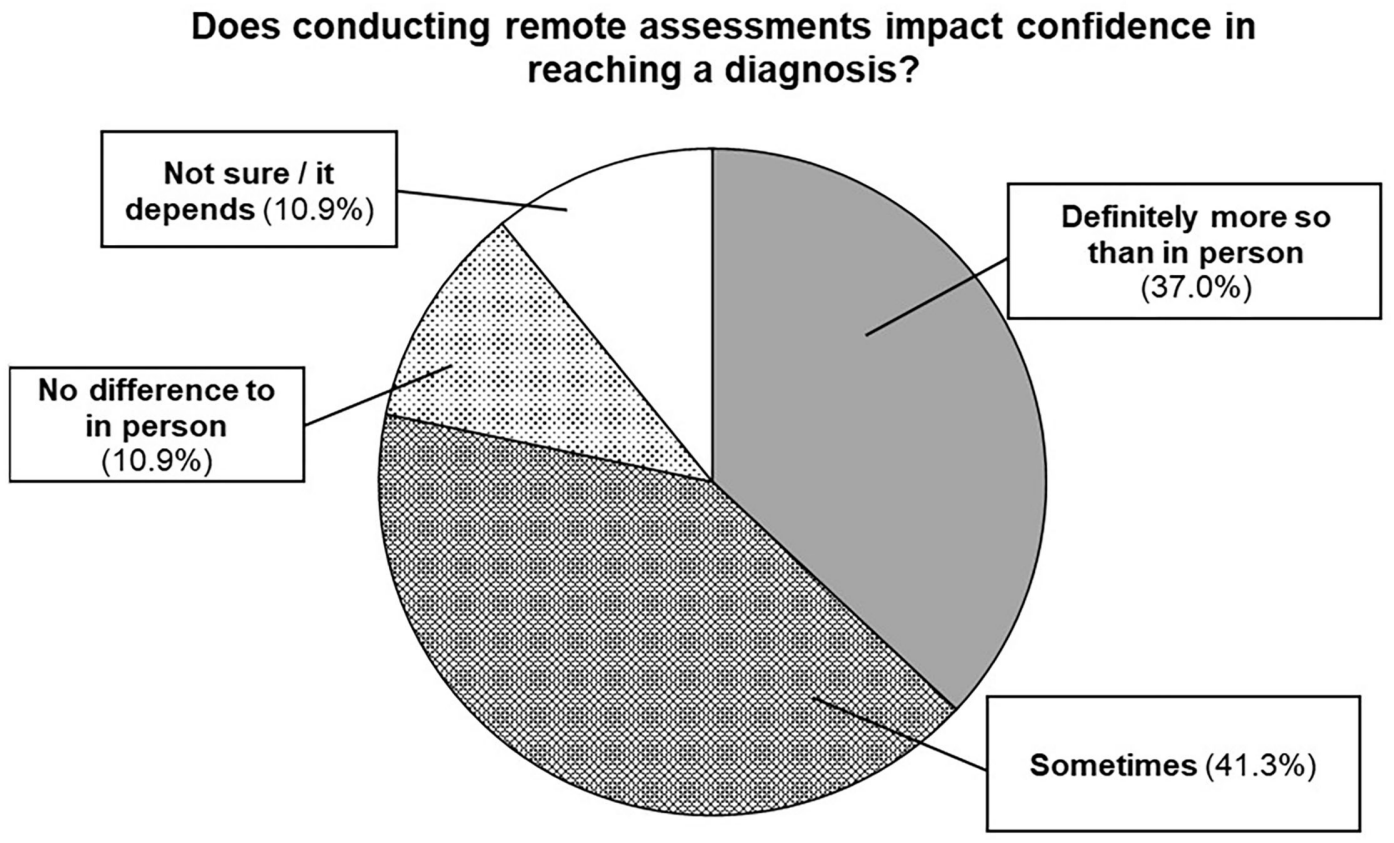
Confidence in diagnosing autism remotely.

Another considered telehealth “*has taken away some clinical observations but provided others at the same time.”* Some participants said there was “*greater uncertainty about [the] likelihood of getting a clear diagnosis,”* and 77% said they had not been able to reach a diagnostic conclusion *via* telehealth in some instances (see Figure 4).

Several steps were taken to reach diagnostic conclusions when using telehealth alone was deemed insufficient. These included: (1) offering more telehealth appointments; (2) involving additional professions; (3) obtaining second opinions, (4) using extra assessment measures (e.g., tests of neuropsychological functioning and/or standardized questionnaires); (5) watching home videos for naturalistic observations; (6) speaking to informants about the patient's behaviors; and (7) if needed, arranging an in-person meeting. Invariably, this meant that patients were placed on an internal waiting list, thus delaying the diagnostic outcome.

#### Theme 4, Subtheme 3: Communicating a Diagnostic Conclusion

A handful of services offered patients remote or in person feedback about diagnosis. Some participants felt communicating a diagnostic conclusion *via* telehealth was much the same as in person. Conversely, others said telling someone they do not have autism remotely, could feel “*tricky,”* especially when patients had waited a long time for the assessment. Participants were mindful “*we might be missing something because we haven't seen them face to face.”* Some patients and families had complained “*if we [health professionals] said it was not ASD then they object as we haven't met them.”*

Conversely, one participant perceived “*all outcomes of an assessment can be equally distressing for a client.”* Not being in the room together meant it was not possible to have “*the same sense of how they [patients] are taking the feedback.”* This was compounded by “*the connection [rapport] with the individual is not the same … [it's] harder to sit with the uncomfortable feelings/disappointment more,”* and it is “*more challenging to manage the emotional impact.”* Some participants provided patients with a “*working diagnosis,”* or a “*don't know outcome;”* to be revisited when they could meet in person. Alternatively, they might delay giving a diagnosis if rapport was poor or instead, offer formulation-led rather than diagnosis-led feedback.

### Theme 5: Improving Service Design and Delivery

The fifth theme pertained to ways in which service design and delivery can be improved. This theme includes five subthemes: (1) understanding that each patient is unique and may require adaptations to telehealth approaches (i.e., no one size fits all); (2) refining new policies and procedures; (3) improvements to service infrastructure; (4) training needs of professionals using telehealth; and (5) the need for innovations for how autism is assessed remotely.

Most participants considered changes to aspects of service design/delivery beneficial. As highlighted by one participant, “*the pandemic is not the cause of waiting list difficulties, decades of austerity and underfunding is. A combination of service redesign (balancing NICE guidelines, clinical quality, and need for brevity/throughput), combined with greater financial investment in staffing is needed. This is the issue, not the pandemic!”*

#### Theme 5, Subtheme 1: No One Size Fits All

Consensus was that different methods of assessment are appropriate for different patients; a combination of in-person and telehealth methods might be pragmatic, if this balanced efficiency and flexibility, with clinical need and risk assessment. Yet some participants had reservations that telehealth might become a panacea, with one describing, “*whilst we will become better at it [remote assessment] with practice, I think we need to sit with the discomfort of a lack of quality of remote autism assessments and very strongly resist allowing commissioners to make this become the 'new normal' forever.”*

#### Theme 5, Subtheme 2: New Policy and Guidelines

Development of new policies and guidelines to outline good/best practice for autism assessment during pandemic conditions was emphasized, including “*how to assess for each of the diagnostic criteria without being face to face … around whether heavier reliance on self-report is adequate and appropriate.” “More robust research”* and building “*an evidence base”* was deemed necessary; focusing on “*the best way to assess remotely,” “efficacy”* of online assessments and “*how remote working might skew assessments of social communication.”* Research into “*the experience and anxieties of the clinicians doing the assessments … [and] the experiences of people receiving diagnosis online*” was also mentioned.

#### Theme 5, Subtheme 3: Infrastructural Change

Infrastructural improvements were deemed a priority. This included better administrative support so that professionals did not spend “*excessive amounts of clinician time”* on non-clinical duties, alongside “*reliable … better technology,” “better [videoconferencing] platforms with less glitch[es],”*, “*access to wifi and devices,”* and “*more tech [technology] support.”* It was also suggested schools and GP surgeries could free up a computer for families with poor or no internet connection. More general suggestions included larger clinic spaces and more equipment for behavioral observation assessments, so that meeting in person might be more viable. Having more time to get to know the patient and develop rapport was also key.

#### Theme 5, Subtheme 4: Training Needs

Participants advocated the need for “*further training”* and “*more practice,”* to enhance IT skills generally and hone capabilities for assessing social communication online. One participant said they would like to “*do more assessments to gain a wider insight.”* Another highlighted it would be helpful to have “*options to watch a gold standard video assessment for a positive diagnosis and no diagnosis, so that we can all be looking for the same sort of service.”* Taken together, participants considered more training, along with “*peer support”* could increase “*confidence.”*

#### Theme 5, Subtheme 5: Innovations

Most participants welcomed “*development”* and “*validation of adapted test batteries;”* specifically, online alternatives to the ADOS-2, IQ tests, and neuropsychological tasks. Building in options to view naturalistic video footage (e.g., home videos), was also considered potentially informative. Some participants thought recording telehealth assessments (with consent), and rating these with colleagues might approximate traditional in person ADOS-2 and ADI-R reliability meetings.

### Theme 6: Post-diagnostic Support

The sixth and final theme pertained to what post-diagnostic support is usually offered. This theme includes two subthemes: (1) the types of support being offered, and (2) how COVID-19 has impacted the provision of this support.

#### Theme 6, Subtheme 1: Types of Post-diagnostic Support

Not all services were commissioned to provide this, but 43 (83%) had offered some form of post-diagnostic support before the pandemic; 26 (50%) of services had offered this during 2020, and another eight (15%) planned to set this up. The types of post-diagnostic support offered varied, including signposting, psychoeducational sessions, family navigation, and less frequently, individually tailored sessions focusing on specific needs (see Table 4).

**Table 4:** Post-diagnostic support interventions.

	**Examples of post-diagnostic support offered**
Clinical support	Follow up appointment to discuss the report and recommendations
	Referral to other services
	Comprehensive assessment of need or aspects of functioning, with appropriate member of MDT
	Social prescribing sessions
	Individual sessions—psychoeducation, occupational therapy, sensory integration, psychological therapies
	Psychoeducational workshops or groups for between one and six sessions, for autistic people—focused on topics including what is autism, social skills, mental health
Community and family support	Family navigator
	Social work support
	Psychoeducational workshops for families, caregivers, and friends
	Parent training
Peer support	Monthly drop-in support groups
	Peer support groups
Vocational support	Employment and benefits advice
	Student mentorship
Information pack and signposting	Resource pack—about autism, related conditions, and options for support locally
	Signposting for patients and/or families

#### Theme 6, Subtheme 2: The Impact of COVID-19 on Post-diagnostic Provision

There had been a move to providing this online or *via* telephone support from Spring 2020. Some, but not all group interventions were now delivered online, making use of options such as “*shared whiteboards and break out rooms.”* Participants stated this “*works for some people but [is] not suitable for all,”* with factors such as patients' preference and risk, influencing what could be offered.

## Discussion

The COVID-19 pandemic has had a fundamental impact on autism diagnostic services in operational and practical terms. Services adapted and responded to the crisis, although the resulting telehealth assessment processes were largely untested in this group. This is one of the first studies to gather perspectives from professionals, working across autism diagnostic services and with patients across the lifespan, about their thoughts about, and experiences of conducting autism assessments before and during the pandemic.

Study findings support the relatively widely held view that there was high demand for diagnostic assessment prior to the pandemic, and unfortunately, a lengthy waiting time for many people (30). Crucially, the onset of the pandemic appears to have exacerbated assessment waiting times. This is clinically concerning, as delays in diagnostic assessment mean that people, and potentially also their families/carers, may not be able to access appropriate needs-led interventions, irrespective of whether they have autism, an alternate condition or both (23, 31, 32).

### Change in Assessment and Staffing Practices Pre-COVID-19 and During the COVID-19 Pandemic

There are likely to be several factors that contribute to lengthy waiting times for diagnostic assessment, including those predating the pandemic; for example, inappropriate or incomplete referrals that require clarification and protract the referral process (e.g., conceivably due to poor knowledge and understanding of autism by health professionals working in primary or secondary care), limited availability of diagnostic services locally or regionally, poor administrative structures, complexities associated with commissioning (e.g., that an individual can only be assessed for one condition at a time, meaning they need a re-referral for an autism assessment when seen by an ADHD diagnostic service that suspects autism is a likely diagnosis) and a mismatch between increasing demand and limited capacity (15, 33, 34). Patient-related factors, such as clinically complex presentations and multimorbidity, may also initially result in mis- or missed-diagnosis (35).

This latter fact may be complicated by our observation that there was variation in the methods used to assess autism across services and age groups. Prior to the pandemic, some, but not all services, incorporated semi-structured interviews (e.g., the ADI-R or DISCO), standardized behavioral observation (e.g., the ADOS-2) and/or formal measures of neuropsychological functioning into the autism assessment, with no participants reporting they conducted assessments purely *via* telehealth. Conversely, during the pandemic, 44 (85%) of participants reported that their service had adapted standard practice, with fewer participants using formalized measures of behavior and/or neuropsychological functioning.

There may also be factors related to the pandemic context that have exacerbated waiting times; for example, arising due to stay-at-home mandates, social distancing guidelines, staff redeployment and sickness, widespread disruptions to care pathways and difficulties experienced by people and their families with accessing telehealth (18, 23, 25). Nonetheless, only 58% of services in our study reported longer waiting times for assessment. This is surprising, given the initial service closures. Further exploration of this result may establish whether additional funding was available for employment of professionals as a result of the pandemic, facilitating, for example, waiting list initiatives. Taken together, more service evaluation and clinically-focused research is needed to better understand the range of systemic factors that influence waiting times, with a view to establishing how these can be addressed in policy, commissioning and clinical practice, during as well as following the pandemic. Future studies examining the relative merits and disadvantages of a standardized approach to assessment, using qualitative and quantitative methodologies, also seem pertinent.

Since the time our study was conducted, a limited number of studies [e.g., (21–24)], and commentaries (19, 25), have been published, focusing on autism assessment during the pandemic and outlining examples of adaptations to standard practice along with considerations for professionals. Yet overall, there is still much we do not know about how professionals are conducting autism assessments, which adaptations are more or less common (and considered more or less critical for reaching a diagnostic conclusion *via* telehealth), and whether this impacts on factors such as conversion rates and patient, family/carer and professional acceptability and satisfaction with service provision.

It may be useful for future studies to investigate whether there have been any systematic differences in autism diagnostic practices during the pandemic, according to the age of clinical population seen (e.g., 0–5 years old, 5–18 year olds, and 18 years old +), abilities of clinical populations (e.g., individuals with or without additional learning disability), service type (e.g., regional vs. national service), or setting (e.g., child development center vs. community mental health service). Such studies should also identify when online assessments are not indicated, and post-pandemic, whether there are parts of the clinical assessment that could be conducted online, as standard, if a blended assessment model is adopted.

### Autism Diagnostic Assessment via Telehealth

Professionals taking part in the present study identified both advantages and challenges to conducting autism assessments *via* telehealth. Many participants considered telehealth, to be efficient, less resource intensive, flexible, cheaper, easily accessible for patients with the right home set up and convenient, for all. Although not used commonly before 2020, telehealth in autism services is not a new concept. Systematic reviews of telehealth autism assessments [e.g., (17)] and telehealth assessment and interventions for autistic people [e.g., (36)] highlight this has been examined for 20+ years, with preliminary evidence of feasibility, reliability, effectiveness and satisfaction for different methods of diagnostic assessment (e.g., the “real time” and “store and forward” methods), and assessment and interventions for autistic people (e.g., relating to speech and language therapy, behavioral management, and cognitive behavior therapy).

While the pandemic has posed substantial challenges, this has indirectly conceivably advanced the use of telehealth more swiftly than otherwise might have been the case. As telehealth is preferred by some patients, a logical step may be for autism diagnostic services to offer choice about whether an assessment will be conducted *via* telehealth, in person or with a hybrid approach. That said, there are clearly issues with parity of service provision; for example, findings reported here and elsewhere [e.g., (18, 22, 23)] indicate telehealth may not be offered to all (e.g., due to clinical presentation or risk), or cannot be offered to all (e.g., due to a lack of internet access). Further evaluation is therefore needed, such as with quality improvement projects, qualitative interviews or focus groups, to establish how to enhance accessibility.

Challenges identified in this study included a lack of parity with regard to accessibility (e.g., that patients with particular profiles were typically expected to wait for in person assessment), IT-related issues, concerns about deviating from usual practice and difficulties assessing autism, patient-related factors (e.g., patients preferring to be seen in person, or having limited privacy at home) and professional-related factors (e.g., difficulties juggling work with home life, feeling less supported with managing risk and clinical decision-making). It is also not feasible to undertake any medical investigations *via* telehealth. Similar findings have been identified in studies conducted during the pandemic with autistic adults', parents'/carers', and/or health professionals' [e.g., (23, 24)].

A very recent review of the impact of the pandemic on different aspects of daily life for autistic people highlighted challenges with telehealth autism assessments, in particular, unreliability of IT and a lack of parity of provision for people who lack the financial means, resources or access, to the internet (37). More research is needed to clarify potential challenges with conducting telehealth autism assessments, from the perspectives of all stakeholders (i.e., patients, families/carers, professionals, and commissioners). Study designs using qualitative methods to obtain in depth data from individual interviews, as well as quantitative methods to obtain cross-sectional data such as *via* a survey, may help to address these research aims.

While advantages to telehealth autism assessments were described by many, participants here also reported polarized views about the validity and reliability of this assessment format. Our survey found that only a proportion of participants (35%) had been meeting patients in person since the pandemic onset. Moreover, the pandemic context had resulted in some practitioners conducting autism assessments as sole diagnosticians, even if the traditional model in their service had involved an MDT approach. Hence, for some, meeting patients remotely proved sufficient to be able to reach a diagnostic conclusion confidently. Others considered this highly inappropriate, unethical and they expressed concerns about being able to form a robust diagnostic conclusion. Our dataset does not fully explain why views are conflicting. However, it is feasible that there are several reasons for this; for example, this may relate to professionals' experience and expertise (e.g., of assessing autism, neurodevelopmental conditions, and general mental health), the amount of training they have completed (e.g., in autism assessment prior to and during the pandemic), confidence in using IT, the nature of referrals a service accepts (e.g., straightforward vs. complex clinical presentations), the diagnostic approach a service uses and whether this has changed because of the pandemic (e.g., professionals working and making a diagnostic decision alone vs. as part of an MDT), service-related trends regarding diagnostic conclusions for patients presenting with complexity (e.g., when there is an absence of corroborative information), whether the assessment incorporates any standardized measures (e.g., an ADI-R) and the amount of support professionals are provided with (e.g., frequency of good quality clinical supervision). Polarization of views could also conceivably be related to professionals' conceptualisations of “autism,” such as whether they view symptoms categorically or dimensionally (2), and their thoughts about what constitutes “impairment.” Moreover, the fact that many professionals are offering an adapted assessment, rather than one that is evidence-based, may also have contributed to unease.

To our knowledge, no prior research has focused on conflicting views of professionals working in autism diagnostic services, about telehealth. However, relatedly, a systematic review of qualitative research by Howes et al. (38), that focused on the experiences of health professionals conducting autism assessments, found variation in views about barriers and facilitators for diagnosis, the importance of MDT working and use of standardized measures. On balance, it is perhaps unsurprising that opinions about the validity and reliability of telehealth autism assessment differ; for many of us, this represents a new way of working. Future studies should investigate what accounts for differences of opinion about clinical practice, and the degree to which this may, for instance, influence the number of standardized measures used per assessment or conversion rates.

The current study established views relatively early on in the pandemic. Surveying professionals in stages, for example, may provide evidence of changes in practice and in confidence if a service has adapted and made improvements to its new way of working. As there is limited research focusing on the validity and reliability of telehealth autism assessments, more studies that examine inter-rater reliability and test-retest reliability of remote vs. in person methods are crucial.

### Clinical Innovation

Participants identified several steps and innovations that might improve autism assessments. The need for updated clinical guidelines outlining good practice in conducting autism assessments *via* telehealth or hybrid approaches was deemed important. There are several commentaries highlighting suggestions and considerations [see (19, 25)], but more empirical research is likely needed to inform development of these. Interestingly, prior research suggests that professionals may not always follow clinical guidelines for autism assessments [e.g., (7, 9, 10, 38)]; potentially because of a combination of service-related, professional-related and patient-related factors. It may be prudent for future research to clarify the ways in which professionals find clinical guidelines useful and reasons for deviating from these.

Study participants also felt that infrastructural changes are needed, such as relating to streamlining administrative processes and enhancing accessibility and parity of provision. Given that services have largely had to rapidly adapt ways of working, it may be that review of internal processes by autism diagnostic services is prudent if this has not already taken place. Ideally, this should be informed by patient and public involvement. Additionally, services may be limited to the use of specific online platforms for assessment, depending on whether they are public or private health settings. Future studies might focus on the relative value of the different platforms from patient, professional and service perspectives, in terms of accessibility, acceptability and reliability.

Many participants demonstrated an openness to using telehealth, but reported they would benefit from additional skills-based training, supervision, and options to share experiences. Traditionally, many services have run consensus reliability meetings focusing on the administration and scoring of standardized autism measures, including the ADOS-2 (39) and ADI-R (40). Working remotely may make it practically easier for professionals to attend consensus meetings, but some measures are not licensed for remote delivery [e.g., the ADOS-2; (21)]. It may, however, be helpful for services to set up consensus meetings for professionals across teams to rate the clinical interview or behaviors/clinical presentation assessed informally *via* telehealth (i.e., in a pre-recorded assessment), subject to the necessary consent from patients.

While efficiency and reduced demand on resources were identified as potential advantages by participants in this study, future research needs to establish the true cost of telehealth assessments, including hidden or under-reported elements of training, additional reviewing and rating of clinical material. Since participants commented that there had been an increase in complexity of referrals prior to the pandemic, confidence in conducting these assessments face-to-face may have been lower, requiring additional tests or clinical collaboration. However, some clinics with long waiting lists are likely to have had to trial their new telehealth approaches on patients who had waited many months, if not years, and hence may have developed a range of other conditions. This is likely to have somewhat impacted confidence in the methods, particularly when the new assessment was likely to be far removed from the standard clinical guidelines and practice.

Finally, most participants reported they would value innovations relating to autism assessment, including development of new assessment measures. It was noted that several participants worked in services that have developed alternative observational assessments to the ADOS-2, although psychometric properties of these are yet to be established. The BOSA is one example of a more formal behavioral observation task designed for use *via* telehealth (21). Yet, this requires facilitation by an adult (i.e., as there are specific prompts and tasks that are needed), and so this is a less feasible method when working with some adults. Further studies investigating the feasibility, reliability, and acceptability of new assessment measures that can be administered online, are clearly needed.

#### Limitations

Several study limitations are noted. The study used a cross-sectional design, meaning that data were collected at one timepoint only. While this is comparable to many studies conducted during the pandemic, this means that we were unable to examine changes over time (e.g., in terms of professionals' thoughts about, or confidence in, telehealth autism assessments). Additionally, data were obtained during the earlier part of the pandemic, and we acknowledge that there may have been changes in practice or thoughts about practice, since that time. As is common for online surveys, we were unable to systematically record the number of potentially eligible people who saw the study flier but opted to not take part; and their reasons for this (e.g., disinterest in the topic, feeling content with service provision before/since March 2020). Similarly, we did not assess whether professionals took part for specific reasons (e.g., strong views about diagnostic processes, positive, or negative experiences of telehealth). The sample was small. Uptake likely could have improved with time, yet we deemed it reasonable to recruit during a relatively brief period given the circumstances and pressures on professionals at that point and uncertainty about a second wave. Some professional disciplines involved in autism assessments were under-represented (e.g., medics, speech, and language therapists), so opinions here may not reflect those of all members of the multidisciplinary team across settings. Some participants were in professional roles that would not generally conduct full autism assessments and be responsible for making diagnoses. In a small sample, this may have impacted on the findings. Most participants were based in the UK so findings may not be generalisable to other countries and settings.

## Conclusions

This preliminary cross-sectional survey study of professionals examined provision of autism assessments prior to, and during the first 6 months of the pandemic. While the sample is small, and further studies focusing on the autism diagnostic pathway during and beyond the pandemic are needed, there were some consistent themes reported by participants. There have clearly been changes to traditional ways of conducting autism assessments. Nonetheless, responses mirror other research findings that the autism diagnostic pathways pre-pandemic were already under significant pressure with increasing demands outstripping capacity. Study participants identified a range of challenges, risks, advantages, and potential opportunities to using telehealth in this context. There was polarization of views about telehealth: some professionals consider this useful and effective; others have significant concerns about whether this is ethical, valid and reliable. Further research—conducted with a range of stakeholders (including patients, partners, carers, and referrers)—is needed to establish more robust, standardized, consistent, accessible, and reliable methods of autism diagnostic assessment for people across the lifespan during and beyond the pandemic.

## Data Availability Statement

The original contributions presented in the study are included in the article/Supplementary Material, further inquiries can be directed to the corresponding author/s.

## Ethics Statement

The studies involving human participants were reviewed and approved by King's College London—[PNM REC REF HR-19/20-17744]. The participants provided their written informed consent to participate in this study.

## Author Contributions

DS, DM, and FH led the design of the study, with contributions from SC, GS, NG, and IE. DS, DM, GS, and SC gathered and organized the data. DS, DM, and GS analyzed and interpreted the data, with input from FH, NG, SC, IE, and JR. DS, GS, DM, and JR wrote the manuscript with contributions from all co-authors. All authors approved the submitted version.

## Funding

FH was part funded by the NIHR Biomedical Research Center (BRC) at the South London and Maudsley NHS Foundation Trust and King's College London.

## Conflict of Interest

JR was employed by Cambridge Lifespan Autism Spectrum Service. NG was employed by Bristol Autism Spectrum Service.

The remaining authors declare that the research was conducted in the absence of any commercial or financial relationships that could be construed as a potential conflict of interest.

## Publisher's Note

All claims expressed in this article are solely those of the authors and do not necessarily represent those of their affiliated organizations, or those of the publisher, the editors and the reviewers. Any product that may be evaluated in this article, or claim that may be made by its manufacturer, is not guaranteed or endorsed by the publisher.
