# Supplementary material for: Autism Diagnostic Assessments With Children, Adolescents, and Adults Prior to and During the COVID-19 Pandemic: A Cross-Sectional Survey of Professionals
Source: Front Psychiatry. 2022 Apr 28;13:789449. doi: 10.3389/fpsyt.2022.789449 (PMC9096098; doi:10.3389/fpsyt.2022.789449)
Supplement: Supplementary file 1 [file Data_Sheet_1.pdf]

Supplementary Material 1. Participant Information Sheet and Survey

PARTICIPANT INFORMATION SHEET - version 1, 30.07.2020

King's College Research Ethics Committee Ref: MRA-19/20-20592

Title of study

Assessment and diagnosis of autism during the COVID-19 pandemic: The ASSESS study

Why have I been invited to take part?

We invite you to take part in this research project. Before you decide if you will take part or not, please read the following information carefully and discuss it with others if you wish. Ask if there is anything that is unclear or if you would like more information.

What is the purpose of the study?

Autism is a common neurodevelopmental condition. Access to an autism diagnostic assessment varies enormously between settings and services. Many children and adults wait months, if not years, for an initial appointment. There are also disparities in the structure and process of diagnostic assessments. Some professionals reach a diagnostic conclusion following a clinical interview; others include standardised semi-structured measures. Provision of post-diagnosis support is patchy, at best, and some people struggle to access support. The COVID-19 pandemic has impacted autism assessment services. Some have temporarily ceased accepting new referrals and seeing clients, whereas other services have adapted this for remote delivery.

This study aims to investigate professionals' experiences of, and perspectives about: (1) how autism diagnostic assessments and post-diagnostic support were offered to children and adults, prior to the COVID-19 pandemic; (2) ways in which assessments and post-diagnostic support have been adapted, as a result of the pandemic (e.g., due to lockdown and social distancing guidelines); (3) what factors make it difficult for people to access a timely, evidence-based diagnostic assessment; and (4) what might improve access during and after the pandemic.

What will happen if I take part?

You will complete an anonymous survey online, that may take 15 - 20 minutes to complete. The first part of the survey will involve reviewing the information sheet. If you decide to take part, you will then give your consent. Next, you will be asked for some general information about what your professional role is, the setting you work in and how long you have worked with autistic people. You will then be asked to note down how autism diagnostic assessments and/or post-diagnostic support were delivered by your service, before the pandemic. We will also ask you about how you think waiting times for an appointment and the process of assessment could be improved, if at all. Finally, you will be asked for your thoughts about, and

experiences of, conducting diagnostic assessments and/or providing post-diagnostic support since the pandemic started (i.e. since lockdown and social distancing guidelines have been in place).

You will need to complete all the survey questions at the same time as your answers will not be saved.

If you meet the following inclusion criteria, we would like to invite you to take part in this research: You are a professional (aged 18 years old and over), involved in conducting autism diagnostic assessments, for children or adults, in any service, setting or country. You use English sufficiently proficiently to complete an online survey.

Do I have to take part?

Participation is voluntary. You should only take part if you want to. Not participating will not disadvantage you in any way. Once you have read the information sheet, contact us if you have any questions that would help you to decide whether to participate. If you do take part, we will ask you to give consent via the online survey.

Incentives

If you would like, your name will be entered into a prize draw. Only validly completed surveys can be entered into the prize draw (to avoid prizes going to bots). Once we have stopped recruiting to the survey (on the 13 September 2020), one person will receive a £50 UK Amazon git voucher, and four people will receive a £25 UK Amazon gift voucher.

What are the possible risks of taking part?

The risk involved in participating is minimal. However, if you have any concerns or worries about the study, you can contact the research team at Dr Debbie Spain, debbie.spain@kcl.ac.uk.

What are the possible benefits of taking part?

You are unlikely to directly benefit from the study. Yet, participating in this will be very valuable for enhancing our understanding of how the waiting time for a diagnostic assessment, and the process of conducting this can be improved, both currently and after the pandemic.

Data collection and handling and confidentiality

Your data will be processed with full confidentiality in accordance with the General Data Protection Regulation 2016 (GDPR). We will store your data in a password protected database on the KCL server. You will be asked to create a unique study identifier that you can remember (instructions are further on in the survey). We will use this as your study identifier. This means no-one will be able to identify you from the research data. At no point will we identify any participant in any report that is produced from this study.

We will retain the data you provide for the duration of the study, and for an additional seven years. We will archive the data electronically; we will keep password protected data sets as outlined above.

During the course of the study, only the research team will have access to the data. Future studies that

examine similar research questions may benefit from having access to your anonymised data. You can give consent on the consent form if you are willing to share your anonymised data with others.

Data Protection Statement

Your data will be processed in accordance with the General Data Protection Regulation 2016 (GDPR). If you would like more information about how your data will be processed in accordance with GDPR please visit the link below: https://www.kcl.ac.uk/research/support/research-ethics/kings-college-london-statement-on-use-of-personal-data-in-research

What if I change my mind about taking part?

You are free to withdraw at any point of the study, without having to give a reason. Withdrawing from this will not affect you in any way. If you choose to withdraw from the study, we will not retain the information you have given thus far. After taking part, you are able to withdraw your data from the project up until 20 September 2020. After this date, withdrawal of your data will no longer be possible as we will have put all anonymised data together for analysis.

How is the project being funded?

There is no particular funding attached to this study.

What will happen to the results of the project?

We will write up the results in a report for lay and academic audiences, and may present the findings at meetings or at conferences. We will seek your consent to use anonymised comments in presentations and publications. After study completion, the anonymised data will be archived for seven years before being permanently deleted. Within this time, if any other research questions relating to this project emerge, we may use the archived data subject to approval from the appropriate ethics committee.

Who should I contact for further information or if something goes wrong?

If this study has harmed you in any way, you can contact King's College London using the details below for further advice and information: Chair of the PNM RESC at rec@kcl.ac.uk. If you have any questions or require more information, please contact us at:

Dr Debbie Spain (Visiting Postdoctoral Clinical Researcher)
Social, Genetic and Developmental Psychiatry Centre
Institute of Psychiatry, Psychology & Neuroscience
King's College London
de Crespigny Park, Denmark Hill
London, SE5 8AB
debbie.spain@kcl.ac.uk

Thank you for reading this information sheet and for considering taking part in this research.

Consent Form

I confirm that by consenting to participate in this study I am agreeing with the following statements:

1. I confirm that I have read and understood the information sheet dated [VERSION 1 30.07.2020] for the above study. I have had the opportunity to consider the information and ask questions which have been answered to my satisfaction.
2. I understand that I must only take part if I meet the inclusion criteria detailed in the information sheet.
3. I consent voluntarily, to be a participant in this study and understand that I can refuse to answer questions and can withdraw from the study at any time, without having to give a reason, up until 20 September 2020.
4. I consent to the processing of my personal information for the purposes explained to me in the Information Sheet. I understand that such information will be handled in accordance with the terms of the General Data Protection Regulation.
5. I understand that my information may be subject to review by responsible individuals from King's College London for monitoring and audit purposes.
6. I understand that confidentiality and anonymity will be maintained, and it will not be possible to identify me in any research outputs.
7. I consent for the research team to use non-identifiable data in future research. I understand that any use of identifiable data would be reviewed and approved by a research ethics committee.
8. I understand that completion of this consent form with the online survey is the process by which I give my consent.

Please pick a consent option:

- o I have read the information sheet, understood the aims and purpose of this study, agree with statements 1-8 above, and I provide my consent to participate.
- o I do not consent to participate in this study.

Study identifier

We will not be collecting any personal details during this study (e.g., your name, address).

Please create a unique identifier code so you have a way of identifying yourself should you need to contact the research team. Please write down this code for later use.

(Open text box)

<u>Section 1</u>

The first few questions of the survey focus on demographic information about your professional background, years of experience working with autistic people, and work setting.

Please select which of the following best describes you. Please select all that apply.

- Psychiatrist
- Paediatrician
- Clinical psychologist
- Counselling psychologist
- Educational psychologist
- Forensic psychologist
- Registered nurse
- Speech and language therapist
- Social worker
- Occupational therapist
- Health visitor
- Teacher
- Assistant psychologist or neurodevelopmental worker / specialist
- Postgraduate researcher
- Postdoctoral researcher
- Academic (professor, lecturer)
- Other (please provide more information) *(Open text box)*

What country do you work in?

(Open text box)

For how many years have you worked with autistic people, for the majority of your professional time?

- Up to 4 years
- 5 – 9 years
- 10 – 14 years
- More than 15 years

What setting do you currently work in? Please select all that apply.

- Autism assessment (diagnostic) service for people living in the local area
- Specialist service for people with intellectual disability (e.g., a mental health in learning disability team)
- Community mental health team (e.g., child and adolescent mental health service, or community mental health team)
- Children’s health centre
- General practitioner (GP) practice
- Inpatient ward
- National specialist (tertiary) service
- School
- Occupational health service
- Residential home or unit
- Health centre at a university (e.g., a service for students and staff)
- Private hospital
- Independent practice (working as a sole practitioner, or as part of a clinic)
- Criminal Justice System
- Other (please provide more information) *(Open text box)*

Is this a public or private service?

- Public
- Private
- I work in both public and private services
- Other (please provide more information) *(Open text box)*

Which age range best describes the people you work with?

- Children and adolescents
- Adults
- People across the lifespan

What clinical disciplines are represented in your service? Please select all that apply.

- Psychiatry
- Medicine
- Psychology
- Nursing
- Speech and Language Therapy
- Occupational Therapy
- Social Work
- Education
- Other (please provide more information) *(Open text box)*

Who can refer people to your service? Please select all that apply.

- Self-referral
- Referred by family or carer
- General practitioner (GP, family doctor)
- Health professional
- Teacher or educational psychologist
- A clinical commissioner or someone who has responsibility to agree to assessments on behalf of the health authority or commissioning service
- Other (please provide more information) *(Open text box)*

<u>Section 2</u>

The following questions focus on how autism diagnostic assessments were offered in your service, prior to the pandemic.

If you participate in assessments in more than one setting (e.g., in the NHS and independent practice), please complete the following questions based on your experience in the service you work in most, and indicate here which service your answers relate to:

(Open text box)

Prior to the pandemic, approximately how many referrals did your service receive per month?

(Open text box)

Prior to the pandemic, approximately how many diagnostic assessments did your service conduct per month?

(Open text box)

Q3.5 Prior to the pandemic, did your service have a triage process for determining eligibility for a diagnostic assessment?

- No
- Yes (if so, please provide brief information about what this involved) *(Open text box)*

Q3.6 Prior to the pandemic, what was the average waiting time (in weeks) for a diagnostic assessment at your service?

(Open text box)

Prior to the pandemic, were referrals ever expedited (i.e., people were seen without having to wait the expected time)?

- No
- Yes (if so, please provide brief anonymised and non-personally identifiable examples of reasons for this, e.g., the diagnosis required clarification as part of a court case, or this was needed to aid decision-making about suitable accommodation following a hospital admission) *(Open text box)*

Q3.8 Prior to the pandemic, how many professionals typically participated in each diagnostic assessment?

- One
- Two
- Three
- Other (please provide more information) *(Open text box)*

Prior to the pandemic, what methods were routinely incorporated into the diagnostic assessment at your service. Please select all that apply.

- Clinical / psychiatric interview with the person
- Triple AAA assessment
- Diagnostic Interview for Social and Communication Disorders (DISCO)
- Autism Diagnostic Observation Schedule (ADOS-2)
- Autism Diagnostic Interview-Revised (ADI-R)
- Interview with a family member, partner or friend
- Developmental, Dimensional and Diagnostic Interview (3di)
- Assessment of IQ (e.g., WISC, WAIS, WASI)
- Assessment of neuropsychological functioning (e.g., theory of mind, executive functioning; please provide more information) (*Open text box)*
- Sensory assessment
- Formal assessment of functioning
- Self-report questionnaires of autism (please provide more information) *(Open text box)*
- Other self-report questionnaires (please provide more information) *(Open text box)*
- Informant-rated questionnaires (please provide more information) *(Open text box)*
- Physical health assessment (please provide more information) *(Open text box)*
- Other (please provide more information) *(Open text box)*

Prior to the pandemic, approximately how many hours did one assessment take? (please add together the total number of hours from each professional who participated in the assessment)

(Open text box)

Prior to the pandemic, did your service provide post-diagnostic support?

- No
- Yes (if so, please provide brief details about what this comprised, e.g., one follow up appointment with a health professional to discuss the recommendations, or a series of group sessions?) *(Open text box)*

With hindsight, what problems did you or your service encounter with regards to providing diagnostic assessments before the pandemic, and what do you think could have helped improve service delivery?

(Open text box)

<u>Section 3</u>

The following questions focus on your thoughts about, and experiences of, participating in autism assessments since the onset of the pandemic.

Was your service temporarily closed due to the pandemic?

- No
- Yes (if so, for how many weeks?) *(Open text box)*

Was the waiting list temporarily closed to new referrals?

- No
- Yes (if so, for approximately how many weeks?) *(Open text box)*

Do you think there has been a change in the referral rate to your service, since February 2020?

- The service seems to have received fewer referrals than before
- The referral rate seems about the same as before
- The service seems to have received more referrals than before

---

Q4.7 How does the current waiting time for an appointment compare to the waiting time prior to the pandemic?

- Waiting time is shorter
- Waiting time is approximately the same as before
- Waiting time is longer

Please use the space below to provide your thoughts on what can (or should) be done to improve waiting times under the current lockdown circumstances?

(Open text box)

Has your service conducted diagnostic assessments since the start of the pandemic?

- No
- Not yet, but preparing to
- Yes

Has your service offered any in person diagnostic assessments since the start of the pandemic?

- No
- Not yet, but preparing to
- Yes

Has your service adapted the assessment process, due to lockdown and social distancing guidelines?

- No
- Not yet, but preparing to
- Yes

If so, how has your service adapted the usual diagnostic assessment process?

(Open text box)

What have been the biggest challenges for you and your service, in relation to providing diagnostic assessments since lockdown?

(Open text box)

Please tell us your views about using personal protective equipment (PPE) during assessments?

(Open text box)

Some services are now providing diagnostic assessments remotely (e.g., via Attend Anywhere, Zoom, Skype and Microsoft Teams). Please tell us about your thoughts, and any non-personally identifiable feedback you have had from your clients or their families, about the advantages and limitations of remote diagnostic assessments.

(Open text box)

How confident do you feel conducting diagnostic assessments remotely, compared to in person diagnostic assessments?

5-point Likert:

- Not at all (0)
- (1)
- Quite a bit (2)
- (3)
- A lot (4)

Is there anything that could increase your confidence to conduct these remotely?

(Open text box)

Q4.18 How difficult is it to assess the following during a remote assessment?

	**Not at all (0)**	**(1)**	**Quite a bit (2)**	**(3)**	**A lot (4)**
**Social communication difficulties**					
**Sensory sensitivities and preferences**					
**Rituals and routines**					
**Repetitive behaviours and stimming**					
**Functional impairment**					
**Mental health**					
**Risk (to or from self/others)**					

Does conducting an assessment remotely impact your confidence to reach a diagnostic conclusion?

- No difference to when doing an in person assessment
- Sometimes
- Definitely more so than when doing an in person assessment
- Not sure / it depends

If so, what factors make it difficult to reach a diagnostic conclusion via a remote assessment?

(Open text box)

Have there been times - prior to or since the pandemic started - when you have not been able to reach a diagnostic conclusion?

- No
- Yes (if so, what is the process at your service for completing these assessments?) (*Open text box)*

Please tell us your thoughts about telling someone that they (or their child) do not meet diagnostic criteria for autism, remotely, rather than in person.

(Open text box)

What innovations or new tools / methods do you think could help you with assessment and diagnosis under the current lockdown circumstances?

(Open text box)

In what ways do you think we could improve the quality of remote autism assessments?

(Open text box)

Is your service offering post-diagnostic support currently?

- No
- Not yet, but planning to
- Yes

Please use this space to tell us about the ways in which your service has adapted the usual post-diagnostic support.

(Open text box)

Please use this space to provide more information, if you would like, about your experiences of providing autism assessments prior to the pandemic or currently.

(Open text box)

- End of Survey
